# Alteration of Cholesterol Metabolism by Metformin Is Associated With Improved Outcome in Type II Diabetic Patients With Diffuse Large B-Cell Lymphoma

**DOI:** 10.3389/fonc.2021.608238

**Published:** 2021-06-14

**Authors:** Xiang-Nan Jiang, Yan Zhang, Wei-Ge Wang, Dong Sheng, Xiao-Yan Zhou, Xiao-Qiu Li

**Affiliations:** ^1^ Department of Pathology, Fudan University Shanghai Cancer Center, Shanghai, China; ^2^ Department of Oncology, Shanghai Medical College, Fudan University, Shanghai, China

**Keywords:** metformin, cholesterol, diffuse large B-cell lymphoma, B-cell receptor, type II diabetes

## Abstract

Recent studies have demonstrated the benefits of metformin on patients with lymphomas. B-cell receptor (BCR)-PI3K-AKT pathway-dependent cholesterol synthesis may represent a positive feedback mechanism responsible for the pathogenesis of BCR-dependent diffuse large B-cell lymphomas (DLBCLs). Thus, restriction of lipid synthesis would affect the integrity of lipid-forming membranes and block the BCR signaling pathway. Our *in vitro* findings suggested that the blocking effect of metformin on BCR signaling pathway is possibly exerted *via* blocking the biosynthesis of cholesterol. A retrospective case-control study was subsequently conducted on type II diabetic patients with DLBCL who were on metformin. Metformin was identified to be associated with improved response rate and PFS in diabetic patients and appeared to be an effective therapeutic drug against DLBCL.

## Introduction

Metformin is one of the most common medications for diabetic patients and can enhance insulin sensitivity by decreasing hepatic glucose production and increasing peripheral tissue glucose use (1). In addition to its hypoglycemic effect, recent clinical studies have roused interest in its anticancer role in different malignancies (2–7). There are also a few studies on the role of metformin in hematological malignancies, such as monoclonal gammopathy of undetermined significance and multiple myeloma (8, 9). However, the effects of concomitant metformin use on the clinical outcomes of lymphoma, for example, the most common subtype, diffuse large B-cell lymphoma (DLBCL) patients remain unclear. Limited retrospective or prospective studies have demonstrated inconsistent results (10, 11).

Several studies have explored the possible mechanisms underlying the anticancer effects of metformin, and adenosine 5’-monophosphate (AMP)-activated protein kinase (AMPK) seems to play an important role (12–14). As one of the serine/threonine protein kinase family members, AMPK can be activated by metformin, and is involved in the regulation of almost all metabolic processes in cells. The metabolic regulatory effects of AMPK classically target the liver, fat, and muscle, and its lipid metabolic effects involve increasing energy material by promoting catabolism and reducing material synthesis by inhibiting anabolism (15–17). AMPK downregulation might further facilitate lymphomagenesis, indicating that AMPK activation can potentially be used to treat lymphoma (18).

Recently, updated schemes of the classification of DLBCL have been proposed according to its gene alterations (19–21). Different molecular subtypes of DLBCL may correlate to different mechanisms of pathogenesis, and thus arouse oncologists’ interests of individualized targeted therapy. It is noteworthy that the survival and progression of a substantial fraction of DLBCL appear to be highly dependent on the B-cell receptor (BCR) signaling. For example, there are genetic alterations involving the BCR-dependent NF-κ B signaling pathway in the MCD and BN2 subtypes of DLBCL, and involving the PI3K pathway in the ST2 and EZB subtypes (19–21). In addition, three subgroups of DLBCL have also been proposed by Monti et al. based on their transcriptional profiles, including the BCR, oxidative phosphorylation (OxPhos), and host response (HR) subtypes (22). In those BCR-dependent DLBCL cases, the activated BCRs are mobile, aggregated, and polarized *via* lipid rafts on the cell membrane surface to amplify the signal transduction effect (23, 24). Lipid rafts-consist of lipids (such as cholesterol), which are essential for this BCR function by maintaining the integrity of lipid rafts (25–27).

BCR is important for the survival of DLBCL, and blocking the BCR signaling pathway may thus inhibit tumor cell proliferation. There remains a question if BCR signaling, or, the metabolism of cholesterol in BCR-dependent DLBCL cells, is related to the metformin-induced activation of AMPK? We thus explored the effects of metformin on the BCR signaling and cholesterol synthesis in DLBCL cell lines first, and then compared the outcomes of diabetic DLBCL patients on metformin with those on other hypoglycemic agents.

## Materials and Methods

### Cell Experiments

Three BCR-dependent (with surface Ig) DLBCL cell lines, OCI-LY1 (IMDM, GIBCO), OCI-LY8 (IMDM, GIBCO), and NU-DUL-1 (1640, GIBCO), were employed. All of the cells were maintained at 37°C in a 5% CO_2_ incubator. The status of surface Ig in the cell lines was provided by American Tissue Culture Collection and Deutsche Sammlung von Mikroorganismen und Zellkulturen GmbH and was validated by immunophenotyping. For BCR stimulation, or cross-linking, 3×10^6^ cells were treated with 10 mg/mL rabbit anti-human IgM (Jackson ImmunoResearch Laboratories, West Grove, PA) or goat anti-human IgG (Jackson ImmunoResearch Laboratories) for 10 min. The expression of two BCR signaling-related molecules, phosphorylated SYK (pSYK) and phosphorylated AKT (pAKT), and a key enzyme for cholesterol biosynthesis, 3-hydroxy-3-methylglutaryl-CoA synthase1 (HMGCS1), was evaluated by immunoblotting. BCRs (Cy3-conjugated AffiniPure F(ab’)2 Fragment Rabbit Anti-Goat IgG or IgM, Jackson) and membrane cholesterol (filipin staining kit; GENMED) were further detected by an immunofluorescence assay. The cells were visualized using a Leica SP5X confocal microscope. Images were captured and analyzed using LAS AF software (Leica Microsystems CMS GmbH). And the BCR and membrane cholesterol levels were evaluated by flow cytometry (FACS Canto II, BD Biosciences) using cell suspensions (5×10^6^ cells each). The extracellular cholesterol was quantified by gas chromatography mass spectrometry (GC/MS) using extracellular culture medium obtained by centrifugation. Cell proliferation activity was assessed using a cell counting kit (CCK-8) (Dojindo, Kumamoto, Japan) according to the manufacturer’s instructions.

### Clinicopathological Studies

The studies involving human participants were reviewed and approved by the Medical Ethics Committee of Fudan University Shanghai Cancer Center. Fifty DLBCL patients with type II diabetes who received metformin throughout their chemotherapeutic treatment course were identified. We extracted important clinicopathological features, including the age, gender, stage of the disease, International Prognostic Index (IPI) score, cell-of-origin (COO) subtype of the tumor, and the outcome of treatment. DLBCL patients with type II diabetes treated with other hypoglycemic agents during the same period, but with other clinicopathological characteristics comparable, served as the control. All of these patients were treated with a regimen containing rituximab, cyclophosphamide, adriamycin, vincristine, and prednisone (R-CHOP). In order to further verify the findings by *in vitro* experiment, we also evaluated the expression levels of pSYK, pAKT, and HMGCS1 in selected cases of both cohorts by immunohistochemistry using formalin-fixed, paraffin-embedded tumor specimens obtained prior to the immunochemotherapy.

### Statistical Analysis

Survival was determined from the time of diagnosis until the time of death (or last follow-up) or progression. Survival curves were constructed by the Kaplan–Meier method. Survival distributions were compared using the log-rank test. All of the statistical analyses were carried out using GraphPad Prism, version 7.0. Categorical variables were compared using the chi-squared test or Fisher’s exact test, and continuous variables were compared with the t test or the rank-sum test. All *P*-values were two sided, and a *P*-value ≤ 0.05 was considered to be statistically significant.

## Results

### Cell Experiments: Metformin May Inhibit the Growth of DLBCL by Blocking the BCR Signaling and the Biosynthesis of Cholesterol

**Figure 1:**
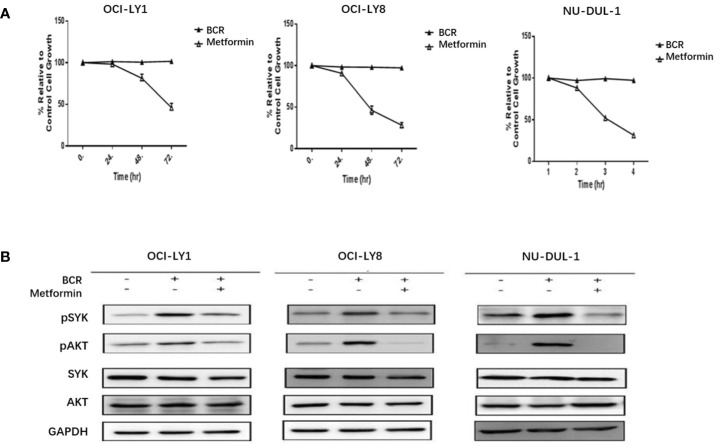
Metformin inhibits the growth rates of three DLBCL cell lines **(A)**. BCR stimulation upregulates the expression of pSYK and pAKT, which is ablated by a pretreatment with metformin **(B)**.

**Figure 2:**
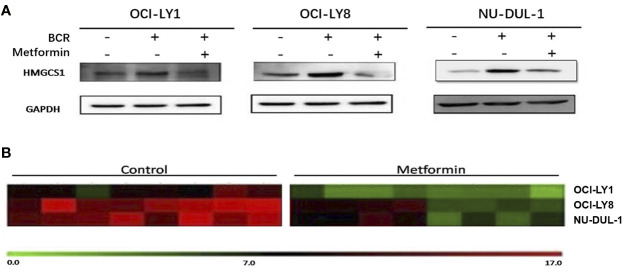
BCR stimulation induces an increased expression of HMGCS1, which is ablated by a pretreatment with metformin **(A)**. The GC/MS detection demonstrates that metformin treatment can reduce extracellular cholesterol concentration **(B)**.

**Figure 3:**
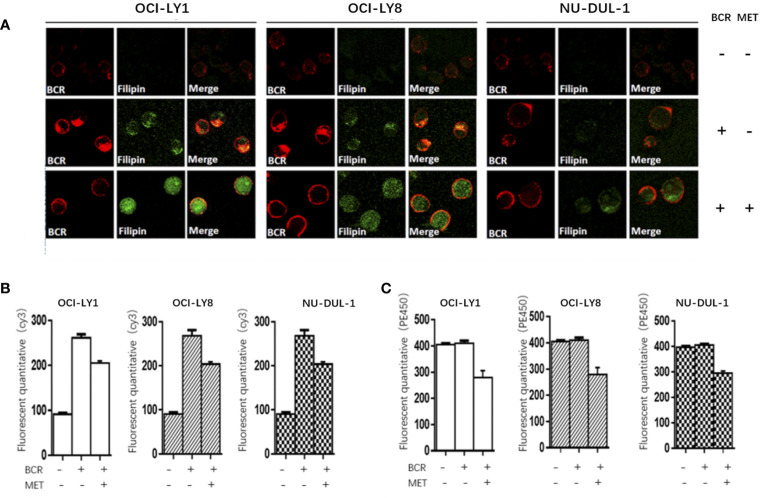
At BCR stimulation, the BCR capping signals (red) and cholesterol capping signals (green by Filipin) appear in three DLBCL lines, and the merged images show the colocalization of these signals. These capping signals decrease or disappear following a pretreatment of metformin **(A)**. The Cy3 (BCR) levels increase after a BCR stimulation, and decrease following a pretreatment with metformin **(B)**. The metformin treatment results in reduced PE450 (membrane cholesterol) levels **(C)**.

*In vitro*, we observed metformin-induced growth inhibition of the tumor cells in all three DLBCL cell lines (**Figure 1A**). The BCR stimulation led to an increased expression of pSYK and pAKT. These activating effects, however, were remarkably ablated by a metformin pretreatment (**Figure 1B**). BCR stimulation also correlated with an increased expression of HMGCS1, which could be ablated by metformin pretreatment, too (**Figure 2A**). The GC/MS detection demonstrated that a pretreatment with metformin could reduce the extracellular cholesterol concentration (**Figure 2B**). By immunofluorescence, we noticed the phenomenon of BCR capping, which was caused by BCR stimulation, and represented polymers formed by BCR cross-linking. The colocalization of the cholesterol and BCR signals was observed throughout the experiment, that is, colocalized cholesterol and BCR capping signals appeared at BCR stimulation, whereas the capping signals of both decreased or disappeared following metformin treatment (**Figure 3A**). In addition, the BCR and membrane cholesterol levels, revealed by flow cytometry assay, were also decreased by the pretreatment with metformin after induction by BCR stimulation (**Figures 3B, C**).

### Clinicopathological Findings: Metformin Improves the Response Rates and Survival of Diabetic DLBCL Patients

**Figure 4:**
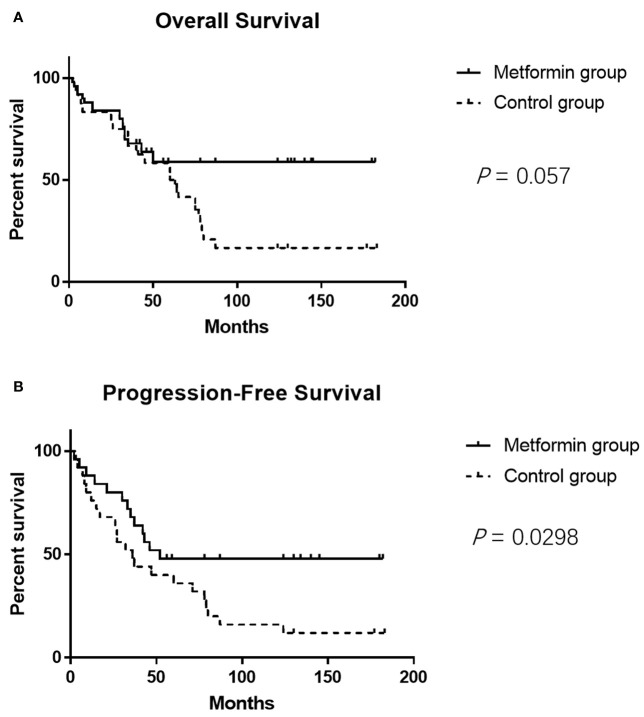
The metformin group shows better PFS compared with the control group (*P* = 0.0298) **(A)**. It seems that the metformin group also features a trend of better OS, but the difference is not statistically significant (*P* = 0.057) **(B)**.

**Figure 5:**
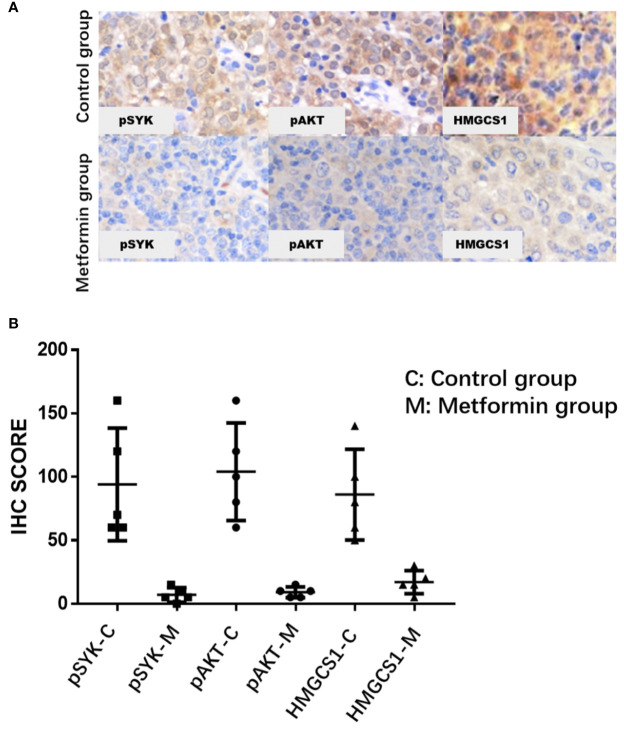
The immunohistochemical expression levels of pSYK, pAKT, and HMGCS1 of DLBCL patients from metformin group are significantly lower than that of control group **(A, B)**.

The clinicopathological characteristics of the DLBCL patients on metformin treatment and the control group were summarized in **Table 1**. In general, there were no significant differences with regard to the distribution of age, gender, stage of the disease, IPI score, and COO subtype of the tumor between the metformin and the control cohort. Of note, patients on metformin showed significantly higher response rates to the immunochemotherapy compared with the control group, reflected by a complete response (CR) rate of 84% and an objective response rate (ORR) of 88% in the former, in contrast to the CR rate of 48% (*P* = 0.0003) and the ORR of 68% (*P* = 0.0283) in the control cohort. The metformin group (with a medium follow-up period of 33 months) also featured a better progression-free survival (PFS) compared with the control group (with a medium follow-up period of 35 months) (*P* = 0.0298). Furthermore, there was a trend of improved overall survival (OS) present in the metformin group, although the difference was not statistically significant (**Figure 4**). Immunohistochemically, the expression levels of pSYK, pAKT and HMGCS1 in the metformin group were significantly lower than those in the control group (**Figure 5**).

**Table 1:** Clinicopathological characteristics of the patients.

Characteristic	Metformin group	Control group	*P*
	*n* = 50	*n* = 50	
**Age** (years),			
median (range)	60 (31-85)	59 (26-86)	0.2038
**Gender**, *n* (%)			
Male	32 (64)	28 (56)	0.5406
Female	18 (36)	22 (44)	
**Stage of disease**, *n* (%)			
I -II	18 (36)	20 (40)	0.8369
III-IV	32 (64)	30 (60)	
**IPI score**, *n* (%)			
0-2	30 (60)	30 (60)	>0.9999
3-4	20 (40)	20 (40)	
**COO subtype**, *n* (%)			
GCB	16 (32)	18 (36)	0.8330
Non-GCB	34 (68)	32 (64)	
**Outcome**, *n* (%)			
CR	42 (84)	24 (48)	0.0003
ORR	44 (88)	34 (68)	0.0283

COO, cell-of-origin; CR, complete remission; GCB, germinal center B-cell-like; IPI, International Prognostic Index; ORR, objective response rate.

## Discussion

DLBCL is a group of biologically heterogeneous diseases, comprising a variety of clinicopathologically distinctive entities, which may need different therapeutic strategies. As aforementioned, Monti et al. demonstrated that DLBCL can be divided into three subgroups, the BCR, OxPhos, and HR, based on their different gene expression profiles (22). Nevertheless, the biological characteristics of these molecular subtypes were poorly understood for a long time, until Caro et al. verified the Monti’s discovery by using two-dimensional electrophoresis at the protein level. They found that mitochondrial oxidative phosphorylation-related proteins are expressed at low levels in BCR-dependent DLBCL (28), which suggests that the energy supply of BCR-dependent DLBCL may depend on anaerobic metabolism. *In vitro* experiments have shown the following metabolic features of BCR-dependent DLBCL: 1) great glucose demand but inefficient utilization; 2) enhanced glycolysis in the cytoplasm and creation of the small molecule “carbon” instead of ATP; 3) low oxygen consumption and highly acidic substance production; and 4) reversal of these phenomena and significant inhibition of cell proliferation upon BCR inhibition (16, 29). Moreover, cholesterol synthesis is remarkably upregulated in BCR-dependent DLBCL, and the raw material for cholesterol synthesis is just the small molecule “carbon” produced by glycolysis (24).

The lipid rafts, composed mainly of cholesterol and glycosphingolipid, are activated to achieve aggregation and polar distribution, which is essential for the signal transduction effect (27). Thus, restriction of cholesterol synthesis will further affect the integrity of cell membranes and influence the biological function of BCRs, resulting in the blockage of BCR signaling pathway. We found that metformin can block cholesterol synthesis by inhibiting the key cholesterol synthesis-related enzyme, HMGCS1, which results in a reduced production of the cholesterol contents, especially that of cell membrane. The decrease of membrane cholesterol leads to an inhibition of cholesterol-dependent BCR and its downstream signaling, that is, BCR signal transduction is blocked at the cell membrane level. However, the mechanism underlying the inhibitory effect of metformin on HMGCS1 in lymphoma cells remains largely unknown. One possibility lies in the roles played by sterol-regulatory element binding proteins (SREBPs), as it has been suggested that SREBPs can be phosphorylated and inhibited by AMPK in liver cells (16, 17, 30).

Few studies have translated the potential benefit of metformin use in DLBCL into clinical applications. A retrospective study showed that diabetic DLBCL patients treated with metformin during first-line immunochemotherapy had significantly improved PFS and OS compared to those of nondiabetic or diabetic DLBCL patients treated with other glucose-lowering agents (11). Another study on the effects of combined metformin and R-CHOP treatment on DLBCL patients, however, did not find any impacts on the response rate, event-free survival, or overall survival (10). Of note, the second study did not balance the clinicopathological parameters, such as the age, stage of disease, IPI score, and COO subtype, which may lead to potential bias, in the study and control groups. To avoid that, we designed the current case-control study to look into the effects of metformin on type II diabetic DLBCL patients by enrolling two cohorts of patients with matched, comparable clinicopathological parameters. The results demonstrated that metformin use indeed contributes to a significantly improved CR rate and ORR, as well as the PFS. Moreover, a trend of better OS was also observed in the metformin group, although the difference did not reach a statistically significant level, possibly due to the relatively short follow-up time. To further verify the effects of metformin on cholesterol-dependent BCR signaling, we also conducted a preliminary *in vivo* study, and obtained the results consistent with that of *in vitro* experiments. Since we employed patients of both cohorts with matched clinicopathological characteristics for the study, and the patients were all under hypoglycemic treatment before receiving biopsy and immunochemotherapy, it’s reasonable to speculate that the more favorable outcome observed in the metformin group could be attributed to the continuous administration of metformin. However, other factors that may lead to potential bias cannot be entirely ruled out. A prospective randomized controlled study is required to further confirm the benefit of metformin on patients with DLBCL.

## Conclusions

We find that metformin may block the BCR signaling by inhibiting the biosynthesis of cholesterol. The agent appears to be an effective therapeutic drug against DLBCL, especially in those BCR-dependent cases. Our preliminary work may provide a novel therapeutic strategy for the care of patients with DLBCL. Clinical trials are essential to evaluate the safety, efficacy, and optimal dosing schedule of metformin combined with immunochemotherapy, especially in nondiabetic DLBCL patients.

## Data Availability Statement

The raw data supporting the conclusions of this article will be made available by the authors, without undue reservation.

## Ethics Statement

The studies involving human participants were reviewed and approved by the Medical ethics committee of Fudan University Shanghai Cancer Center.

## Author Contributions

Formal analysis: X-NJ and W-GW, investigation: X-NJ and YZ, methodology: W-GW and YZ, project administration: X-QL, resources: X-QL, software: X-NJ, writing, review and editing: X-NJ and X-QL. All authors contributed to the article and approved the submitted version.

## Conflict of Interest

The authors declare that the research was conducted in the absence of any commercial or financial relationships that could be construed as a potential conflict of interest.
